# Effect of Calcination Temperature and Strontium Addition on the Properties of Sol-Gelled Bioactive Glass Powder

**DOI:** 10.3390/gels11060401

**Published:** 2025-05-27

**Authors:** Pei-Jung Chang, Jia-Yu Chen, Chi-Han Cheng, Kazuhiro Aoki, Cherng-Yuh Su, Chung-Kwei Lin

**Affiliations:** 1 Graduate Institute of Manufacturing Technology, National Taipei University of Technology, Taipei 106, Taiwan; t110569006@ntut.edu.tw; 2 Research Center of Digital Oral Science and Technology, College of Oral Medicine, Taipei Medical University, Taipei 110, Taiwan; 3 School of Dental Technology, College of Oral Medicine, Taipei Medical University, Taipei 110, Taiwan; b210110001@tmu.edu.tw; 4 School of Dentistry, College of Oral Medicine, Taipei Medical University, Taipei 110, Taiwan; m204113014@tmu.edu.tw; 5 Department of Basic Oral Health Engineering, Graduate School, Institute of Science Tokyo, Tokyo 113-8549, Japan; kazuhiro_aoki.bhoe@tmd.ac.jp

**Keywords:** sol gel, calcination, strontium, bioactive glass, hydroxyapatite, mineralization, biocompatibility, cytotoxicity

## Abstract

Strontium-added bioactive glass (SBG) has been widely used in bone tissue engineering. SBG can be prepared by conventional high-temperature melt-quenching or calcining sol-gelled powder at 700 °C or above. In the present study, the effects of calcination temperature (400–650 °C) and the amount of strontium addition (0–7 mol.%) were investigated simultaneously. The sol-gel process and post-calcination were used to prepare the Sr-added 58S bioactive glass (SBG) powders. The bioactivity of the SBG powder was assessed by immersing it in simulated body fluid, while biocompatibility and cytotoxicity were evaluated using L929 and MG63 cells, and a zebrafish animal model. The calcination temperatures were determined by thermogravimetric analysis based on the weight loss at various stages. X-ray diffraction was used to reveal the crystalline structure of calcined or SBF-immersed SBG powders. Meanwhile, the texture characteristics of SBG powders were examined by the BET method. Fourier-transformed infrared spectroscopy and scanning electron microscopy were used to investigate the absorption bands and powder morphology of SBG powders before and after SBF immersion. The experimental results showed that all SBG powders were mesoporous with a high specific surface area larger than 200 m^2^/g. SBG powder calcined at 650 °C with 5% Sr addition possessed a major Ca_14.92_(PO_4_)_2.35_(SiO_4_)_5.65_ phase, the smallest pore size of 5.86 nm, and the largest specific surface area of 233 m^2^/g. It was noncytotoxic and exhibited good bioactivity and biocompatibility.

## 1. Introduction

As the aging population increases, the number of bone defect cases has risen significantly, driving a high demand for advanced bone repair materials. These materials must be biocompatible, anti-inflammatory, and anti-infective while being biodegradable and capable of promoting bone tissue regeneration. Thus, the development of bioactive glass has become a key focus in current research and innovation. Bioactive glass (BG) was discovered by Prof. L.L. Hench’s team as Bioglass^®^ 45S5 in 1969 [1]. It is composed of 45 wt.% SiO_2_, 24.5 wt.% Na_2_O, 24.5 wt.% CaO, and 6 wt.% P_2_O_5_ [2], and the composition can be bonded chemically with bone tissue to form a hydroxyapatite (HA) layer and promote cell growth [3]. After the invention of Bioglass^®^ 45S5 [1,2], a series of bioactive glasses with various compositions have been investigated, such as 58S [4], 64S [5], 70S [6], 80S [7], etc. Its excellent biological performance has made bioactive glass a superior bone substitute in bone tissue engineering. The main preparation methods for bioactive glass include melt-quenching [8,9], spray drying [10,11], spray pyrolysis [12,13], and the sol-gel process [14,15].

Melt-quenching and sol-gel are conventional techniques to prepare BG powder. By melt-quenching, a mixture of starting powders is mixed, melted at high temperature, and quickly quenched to prepare bioactive glass frits. For energy conservation, the low-temperature sol-gel method is an alternative way to prepare the desired BG powder. The sol-gel process, a facile chemical route, has been widely used to prepare BG powder due to its unique processing advantages. For instance, various precursors for ion sources, adjustable parameters (aging time, calcination temperature, and time), surfactants, and incorporation of therapeutic ions (zinc, strontium, etc.) can be used to modify the so-prepared BG for the desired applications [16]. In addition, the sol-gel process can be used to prepare BG powders, films, and objects with desired shapes. This makes the sol-gel process an attractive processing technique for various applications [17]. Numerous research papers make use of the sol-gel process to prepare bioactive powders that can serve as carriers for drug delivery and additives for bioscaffolds. Doping with various functional ions can further extend the applications of bioactive glass powder. Many biologically active ions, such as zinc, magnesium, cerium, strontium, fluoride, etc., have been incorporated with BG to grant it antibacterial, anti-inflammatory, and osteoconductive properties. Pantulap et al. [18] have reviewed a wide variety of less-common ions that were introduced into the bioactive glass systems. Among the doped ions, strontium ion was the most widely investigated, followed by Zn, Ag, Mg, etc., for the past two decades.

Strontium is abundant in the human body, with the majority found in bones and teeth. Sr is an alkaline earth metal element and is in the same group as calcium, allowing it to easily substitute calcium to form an apatite phase. Its bone-stimulating and antibacterial abilities make Sr an attractive doping element in bioactive glass for bone tissue engineering [19]. Silva et al. reviewed the effects of strontium-incorporated bioactive glass on bone regeneration [20]. The preparation techniques, types of BG, and the amount of strontium concentration have been addressed. Generally, sol-gelled bioactive glass with less than 5% Sr doping exhibits an improvement in the formation of hydroxyapatite. For example, Moghanian et al. [21] evaluated the effect of 700 °C-calcined sol-gel derived 0, 5, and 10 mol.% Sr-doped BG and demonstrated that 5 mol.% Sr-BG exhibited the maximum cell proliferation and alkaline phosphatase activity, as well as antibacterial ability. Ma et al. [4] investigated 58 SiO_2_-(38-*x*)CaO-*x*SrO-4P_2_O_3_ with x ranging from 0 to 10 mol.% and reported that Sr-containing BG decreased the sample degradation and delayed the apatite formation. Goudarzi et al. [22] also studied 58SBG at an interval of 2 mol.% of Sr, and the HA formation rate reached a maximum with 2 mol.% Sr substitution. Another work by Macon et al. [23], who prepared 58S BG (60 SiO_2_-36CaO-4P_2_O_3_, mol.%) by the sol-gel process and used SrO to substitute CaO (0–100 mL.%), demonstrated that HA nucleation did not occur at high Sr concentrations (75 and 100 mol.% SrO). Bahati et al. [14] prepared 74SiO_2_-(26-*x*)CaO-*x*SrO, where x = 0–5, and found that the in vitro apatite-forming ability decreased with increasing Sr concentration. Hu et al. [24] studied 80SiO_2_-4P_2_O_3_-16(SrO: CaO), where SrO = 0, 6, 15 mol.%, and found that 6Sr-BG was a promising dental repair biomaterial. Excess Sr substitution (15 Sr-BG) decreased cell proliferation, differentiation, and mineralization abilities.

As mentioned above, sol-gel-derived bioactive glass materials were calcined at temperatures ranging from 600 to 700 °C. The substitution of calcium ions by various concentrations of strontium ions was widely investigated with different types of bioactive glasses. For instance, Table S1 summarizes a comparison of various sol-gelled 58S bioactive glass powder with Sr additions. It can be noted that the effect of calcination temperature and Sr concentration on the structure, bioactivity, and biocompatibility of bioactive glass has seldom been investigated simultaneously. As shown in Table S1, Moghanian et al. [21] prepared Sr-doped BG (0, 5, and 10 mol.%) by the sol-gel process, and calcination was performed at 700 °C for 3 h. Texture characteristics (specific surface area and pore size), optimal Sr doping around 5 mol., and the effect of calcination at lower temperatures were not investigated. In the present study, bioactive glass powder with various Sr concentrations (3, 5, and 7 mol.%) was prepared by the sol-gel process followed by calcination at relatively lower temperatures (400–650 °C). The effect of calcination temperature and the amount of strontium concentration on the crystalline structure, texture characteristics, bioactivity, and biocompatibility of Sr-BG powders was investigated. Furthermore, selected samples were examined further by using a zebrafish animal model to determine the cytotoxicity before practical application.

## 2. Results and Discussion

### 2.1. Effect of Calcination Temperature and Strontium Addition on SBG

**Figure 1:**
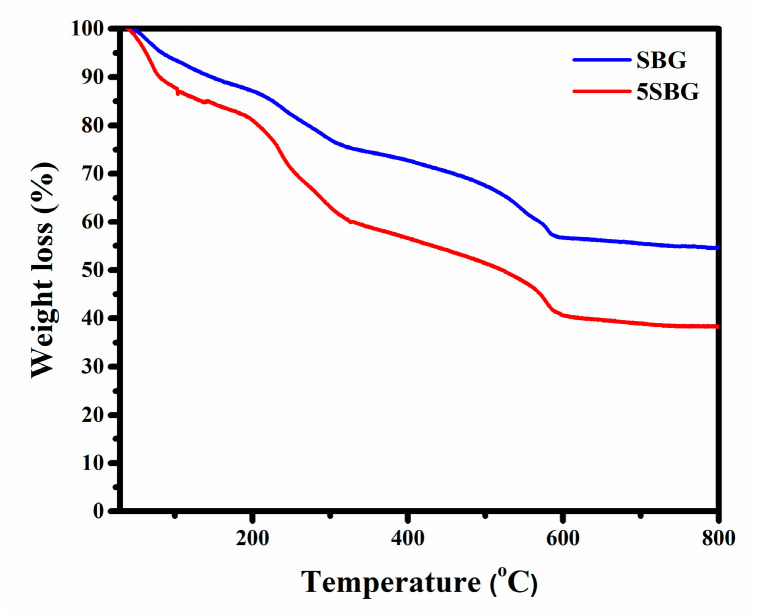
Thermogravimetric analysis of 0SBG and 5SBG powders.

In order to select suitable calcination temperatures, 0SBG and 5SBG as-prepared sol-gelled powders were examined using thermogravimetric analysis, and the results are shown in Figure 1. Generally, the sol-gelled powders exhibited various stages of weight loss before stabilization. For the 0SBG powder (blue curve in Figure 1), a weight loss (6.4%) before heating up to 100 °C was observed. This can be attributed to the vaporization of absorbed water within the powder. Within the temperatures ranging from 100 to 325 °C, another weight loss (18.2%) occurred, probably due to the decomposition of organic solvents. From 325 to 600 °C, organic solvents continued to decompose, and crystallization occurred, which resulted in another stage of weight loss, 18.7%. Thereafter, limited weight loss of ~2% was exhibited for temperatures ranging from 600 to 800 °C. The overall weight loss for 0SBG powder was 45.3% during thermogravimetric analysis from room temperature to 800 °C. A similar trend can be observed for 5SBG powder (red curve in Figure 1), but the magnitude of weight loss (61.7%) was larger when compared to that of 0SBG powder. The weight loss for various stages was 12.2% (RT to 100 °C), 27.2% (100–325 °C), 19.5% (325–600 °C), and 2.3% (600–800 °C), respectively. The increase in weight loss with the substitution of calcium by strontium was also reported in the literature [21,25].

**Figure 2:**
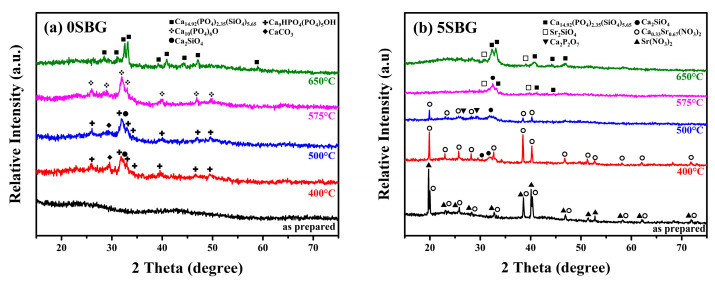
X-ray diffraction patterns of as-prepared and calcined (**a**) 0SBG and (**b**) 5SBG powders.

Based on the thermogravimetric analysis (TGA) results, limited weight loss was observed at temperatures higher than 600 °C. This suggests that calcination at 650 °C was able to remove the harmful organic residues within the bioactive glass powder. Thus, calcination was performed at 400, 500, 575, and 650 °C for 3 h, respectively. The X-ray diffraction (XRD) was used to reveal the crystalline structure of 0SBG and 5SBG powders after calcination. As shown in Figure 2a, the as-prepared 0SBG powder was amorphous without distinct diffraction peaks. After calcination at 400 °C, a few diffraction peaks appeared that corresponded to Ca_9_HPO_4_(PO_4_)_5_OH (calcium deficient hydroxyapatite or CDHAp, PDF 46-0905), Ca_2_SiO_4_ (calcium silicate, PDF 29-0369), and CaCO_3_ (calcium carbonate, PDF 05-0586) phases. A similar diffraction pattern with stronger diffraction intensities was observed for 0SBG powder calcined at 500 °C [26,27]. In addition, decomposition of CaCO_3_ and an increase in calcium silicate were also observed. Ca_2_SiO_4_ became the major phase instead of CDHAp. The complete decomposition of CaCO_3_ and phase transformation of Ca_2_SiO_4_ and CDHAp into oxyapatite (Ca_10_(PO_4_)_6_O, PDF 89-6495) occurred for 575 °C-calcined 0SBG powder [28]. When the calcination temperature was further increased to 650 °C, the calcium phosphate silicate phase (Ca_14.92_(PO_4_)_2.35_(SiO_4_)_5.65_, PDF 83–1494) became the major phase. Table 1 summarizes the detailed percentages of various phases for calcined 0SBG powder.

With the addition of strontium (Figure 2b), the as-prepared sol-gelled 5SBG powder exhibited a mixture of Ca_0.33_Sr_0.67_(NO_3_)_2_ phases (PDF 26-1073) and Sr(NO_3_)_2_ (PDF 25-0746). After calcining at 400 °C, Sr(NO_3_)_2_ disappeared, the crystalline peaks of Ca_0.33_Sr_0.67_(NO_3_)_2_ became more evident, and the formation of Ca_2_SiO_4_ was observed. When the calcination temperature was further increased to 500 °C, partial decomposition of the Ca_0.33_Sr_0.67_(NO_3_)_2_ phase (from 86.0 to 59.0 wt.%) was observed. In addition, a slight increase in the percentage of Ca_2_SiO_4_ (from 14.0 to 25.2%) and the formation of a calcium pyrophosphate (Ca_2_P_2_O_7_, PDF 33-0297) phase were observed [29]. The nitrides were decomposed completely after calcination at a temperature of 575 °C, and the formation of Ca_14.92_(PO_4_)_2.35_(SiO_4_)_5.65_ (PDF 83-1494) occurred [30]. For 650 °C-calcined 5SBG powder, Ca_14.92_(PO_4_)_2.35_(SiO_4_)_5.65_ was the major phase (87.8%), with a minor Sr_2_SiO_4_ (PDF 38-0271) phase [31]. The XRD patterns with detailed diffraction peaks of those referred phases are shown in Figure S1. The relative percentages of various phases for calcined-5SBG powders are summarized in Table 2.

**Figure 3:**
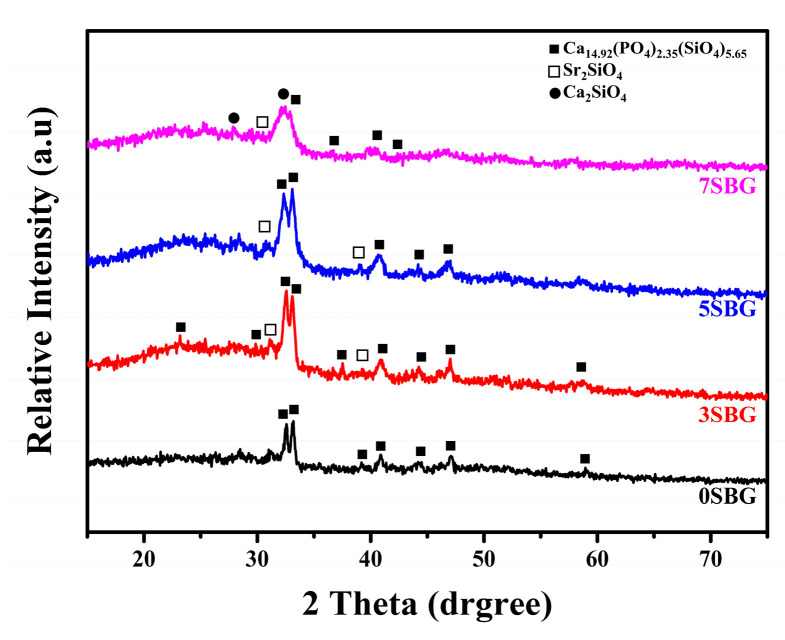
X-ray diffraction patterns of 0–7SBG powder after calcination at 650 °C for 3 h.

For testing the practical usage of bioactive glass, a calcination temperature of at least 650 °C was used in a previous study [21]. Thus, the calcination was set at 650 °C to investigate the effect of strontium concentration. Figure 3 shows 0–7SBG powder after calcination at 650 °C for 3 h. It can be noted that calcium phosphate silicate (i.e., Ca_14.92_(PO_4_)_2.35_(SiO_4_)_5.65_) was the major phase for all 0–7SBG powders, and its percentage decreased with increasing Sr concentration. With the addition of strontium, the formation of Sr_2_SiO_4_ was observed. Figure S2 shows the XRD patterns of 650 °C-calcined 0–7SBG powder with the referred phases. The amount of strontium silicate increased with increasing Sr addition up to 5SBG. For 7SBG with a high Sr concentration, the formation of calcium silicate was observed. Table 3 summarizes the resulting phases and the corresponding percentage of these 0–7SBG powders.

**Figure 4:**
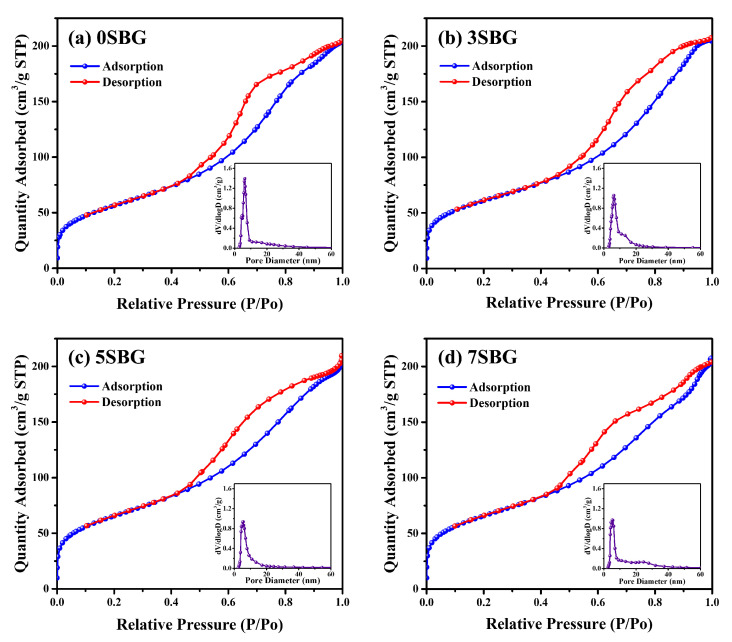
BET analysis of (**a**) 0SBG, (**b**) 3SBG, (**c**) 5SBG, and (**d**) 7SBG powder after calcination at 650 °C for 3 h. The insert in each figure shows the result of pore size analysis.

In addition to the crystalline structures examined using the X-ray diffraction technique, texture characteristics, including surface area and porosity, were determined using the Barrett–Emmett–Teller (BET) method [32]. The specific surface area was 204.31 m^2^/g for 0SBG. It increased to 216.41 m^2^/g for 3SBG, reached a maximum of 233.07 m^2^/g for 5SBG, and slightly decreased to 232.56 m^2^/g for 7SBG. The addition of strontium increased the specific surface area of bioactive glass powder. In addition to the specific surface area, Figure 4 shows the nitrogen adsorption–desorption isotherms of the 0–7SBG powders. Similar hysteresis loops were observed for all 0–7SBG powders that exhibited a Type IV isotherm. It is also noted that the hysteresis loop possessed an inclined mound due to the existence of mesopores and was classified as semi-IUPAC H2 [33]. The average pore diameter was 6.04, 6.39, 5.86, and 5.92 nm for 0, 3, 5, and 7SBG, respectively. The pore size did not exhibit a monotonic trend, though 5SBG had a relatively small one. Moreover, the total pore volume was also similar. It was 0.32 cm^3^/g for 0 and 3SBG, and slightly decreased to 0.31 cm^3^/g for 5 and 7SBG. Table 4 summarizes the results of BET analysis for various 650 °C calcined-SBG powders.

### 2.2. In Vitro Mineralization of SBG Powder

**Figure 5:**
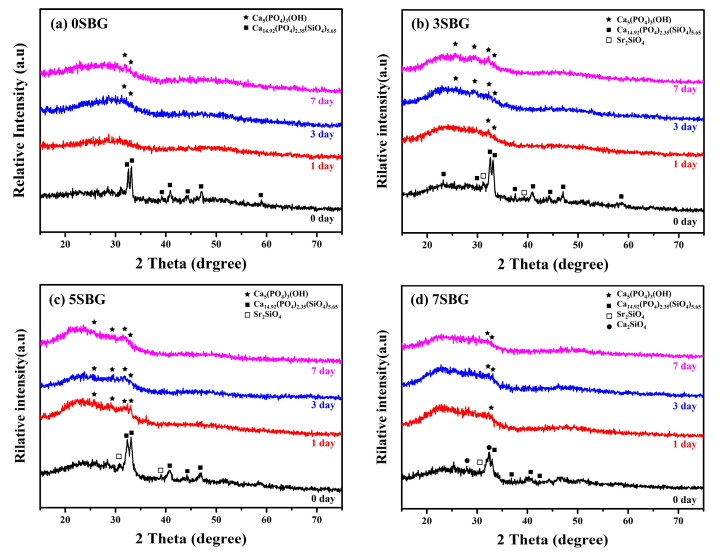
XRD patterns of (**a**) 0SBG, (**b**) 3SBG, (**c**) 5SBG, and (**d**) 7SBG powder after immersion in simulated body fluid for 1, 3, and 7 days.

The bioactivities of the SBG powders were investigated by immersing them in simulated body fluid. The crystalline structure, powder morphology, and functional groups before and after immersion in simulated body fluids (SBFs) were examined using X-ray diffraction, scanning electron microscopy, and FT-IR spectroscopy. Figure 5 shows the XRD patterns of 0–7SBG powders before and after immersion in SBF for 1, 3, and 7 days, respectively. Similar behavior was observed for all SBG powders. After immersion in SBF for 1 day, almost all the crystalline peaks of the SBG powders disappeared (Figure 5a,b) or weakened significantly (Figure 5c,d). This suggests that the calcium or strontium (phosphate) silicates were dissolved into the SBF solution after immersion in SBF. For 0SBG powder with prolonged immersion to 3 or 7 days (Figure 5a), ambiguous hydroxyapatite crystalline peaks (PDF 09-0432) were observed after 7 days of SBF immersion [34]. With the addition of strontium (3SBG, Figure 5b), the formation of hydroxyapatite was observed after 1 day of SBF immersion, and the peak intensities increased with immersion time. This phenomenon became more evident for 5SBG, Figure 5c. The crystalline peaks of HA were the strongest compared to the other SBG powders. The HA phase became uncertain when the Sr concentration further increased to 7%, Figure 5d. The superfluous Sr concentration did not further improve the in vitro bioactivity but hindered the formation of hydroxyapatite. This shows a similar trend to that reported in the literature [4,23]. The crystalline phases of XRD patterns with references before and after immersion in SBF were presented in Figure S3. The SBF immersion experiments suggest that 5SBG powder exhibited optimal in vitro mineralization activity.

**Figure 6:**
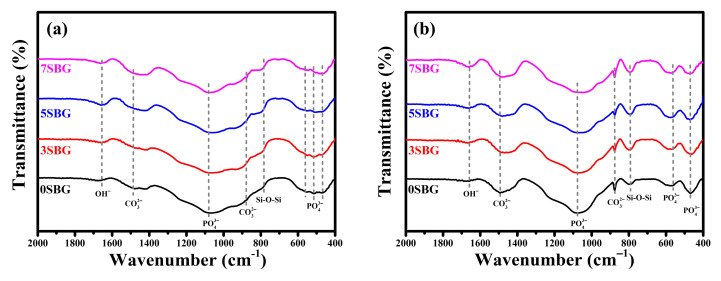
FT-IR spectra of 0-7 SBG powder (**a**) before and (**b**) immersion in simulated body fluid for 7 days.

Figure 6 shows the FT-IR spectra of 0–7SBG powders before and after immersion in SBF for 7 days. As shown in Figure 6a, similar FT-IR spectra were observed for all SBG powders. Relatively broad bands of PO_4_^3−^ at 1073, 559, 517, and 469 cm^−1^ were observed together with CO_3_^2−^ (1490 and 878 cm^−1^), Si-O-Si (783 cm^−1^), and OH^-^ (1654 cm^−1^) bands. After SBF immersion (Figure 6b), these bands became more evident compared to those of the so-prepared SBG powders. The vibration bands of PO4^3−^ at 1076, 563, and 468 cm^−1^ indicated the formation of hydroxyapatite [35,36].

**Figure 7:**
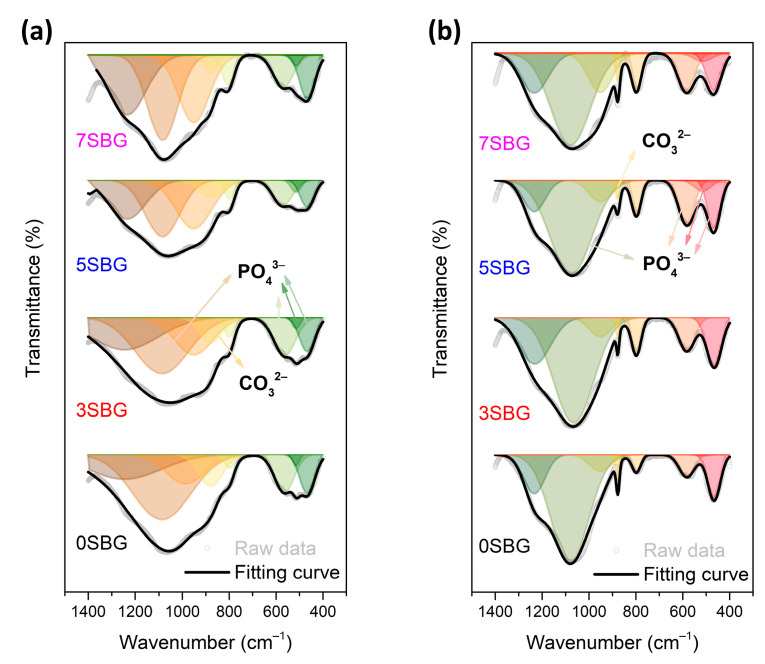
The FTIR deconvolution curves (**a**) before and (**b**) after immersion in simulated body fluid for 7 days.

**Figure 8:**
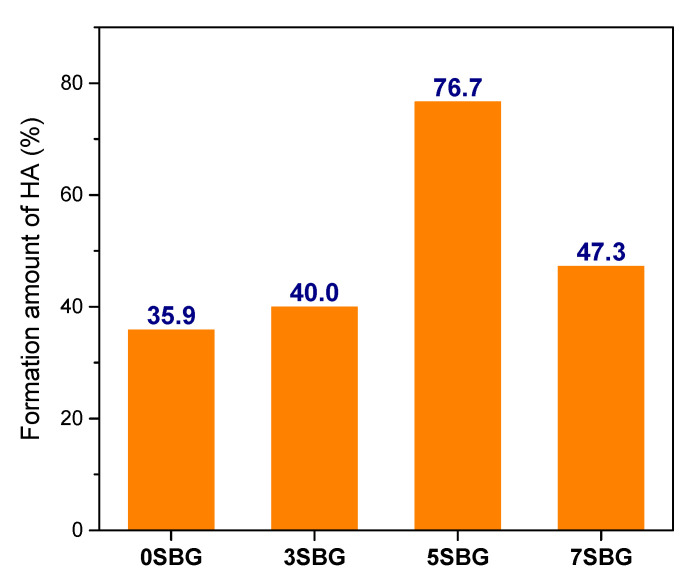
The formation amount of HA calculated from the fitting area of PO_4_^3−^ in FTIR spectra.

The presented FTIR curves were further deconvoluted with the observed bands with the Gaussian method [37,38,39]. The main difference of absorbed bands for HA formation fell within the wavenumber range from 1400 cm^−1^ to 400 cm^−1^. The PO_4_^3−^ functional groups (1073, 559, 517, and 469 cm^−1^), which were the characteristic IR absorption bands of HA, were calculated from their obtained fitting area percentage for semi-quantifying HA formation in SBF. Figure 7a shows the IR deconvolution curves with their fitting area. The estimated amounts of PO_4_^3−^ functional groups were 58.6, 50.2, 43.7, and 44.8% for 0, 3, 5, and 7SBG, respectively. After 7 days of immersion in simulated body fluid, as shown in Figure 7b, the absorbed bands of PO_4_^3−^ grew to 77.2, 70.3, 77.2, and 66.0% for 0, 3, 5, and 7SBG, respectively. The existence of CO_3_^2−^ is able to enhance the HA formation [40,41]. However, the amount of CO_3_^2-^ (878 cm^−1^) decreased after 7 days in simulated body fluid immersion from the FTIR spectra. The results suggest that the OH^−^ in simulated body fluid (pH = 7.4) reduced the amount of CO_3_^2−^ on the SBG powders [42]. The amounts of PO_4_^3−^ and CO_3_^2−^ are listed in Table 5. Furthermore, the HA formation amount was calculated by the difference of the PO_4_^3−^ amount before and after SBF immersion to compare the HA formation ability, and the results are shown in Figure 8. It can be noted that the doping of Sr in bioactive glass improved the HA forming ability, and 5SBG exhibited the highest formation amount of HA (76.5%).

**Figure 9:**
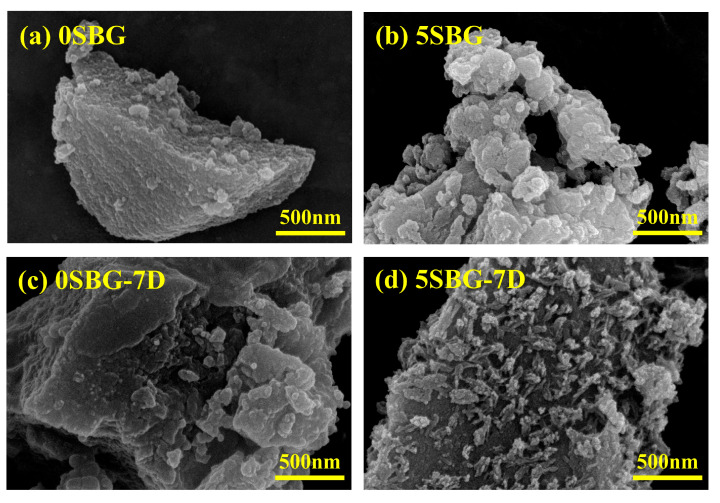
SEM images for 0SBG and 5SBG before (**a**,**b**) and after (**c**,**d**) immersion in simulated body fluid for 7 days, respectively.

The surface topography of the 0SBG and 5SBG before and after SBF immersion was examined by scanning electron microscopy, and Figure 9 shows the corresponding images. Before immersion, the 0SBF powders exhibited a plate-like microstructure covered with some small particles, as shown in Figure 9a. For the 5SBG powder shown in Figure 9b, plate-like particles with smaller particles were observed. After immersing in SBF, repetitive dissolution and precipitation of the SBG powder occurred. For 0SBG powder, precipitation of some small particles was observed, as shown in Figure 9c. This phenomenon became more evident for 5SBG powder. As shown by Figure 9d, numerous particles were precipitated on the large plate particles. Figure S4 shows the elemental mappings with Si, Ca, P, Sr, C, and O elements for 0SBG and 5SBG powders before and after immersion in SBF for 7 days, and Table S2 summarizes the corresponding EDX mapping results. A slight increase in Sr concentration after immersion was noticed. Compared with the FTIR deconvolution results, it was difficult to quantify the HA formation by EDX before and after SBF immersion, probably due to the relatively small amount of HA formation on the SBG powder. In addition to EDX, pH values after various SBF immersion times were measured, and the results are shown in Figure S5. An increase in pH value after immersion was noticed and 0SBG exhibited a slightly larger pH value than 5SBG, probably due to the release of Sr ions into the SBF solution, as shown in Figure S6.

As shown by the XRD, FTIR, and EDX, the deconvolution of FTIR spectra provided semi-quantitative evidence for HA formation after immersion in SBF. The amount of HA formation was 35.9% for 0SBG. It increased with Sr doping, reached a maximum of 76.7% for 5SBG, and decreased to 47.3% for 7SBG. This suggests that 5SBG powder was optimal among all SBG powders [24].

### 2.3. Biocompatibility and Cytotoxicity of SBG

**Figure 10:**
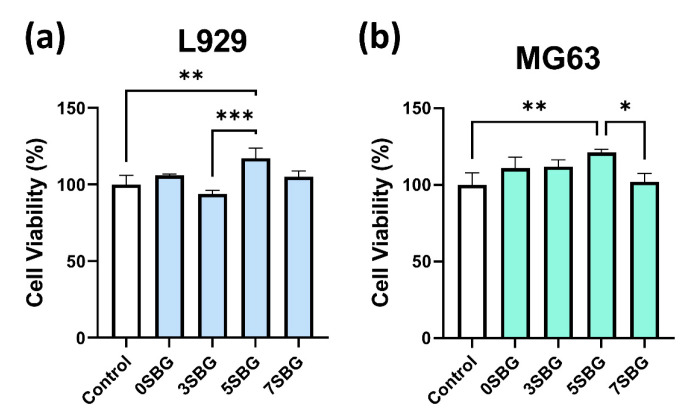
Cell viabilities of (**a**) L929 and (**b**) MG63 for SBG powders were examined by using CCK-8 assay. *, **, and *** indicate that these two samples were statistically different at a 95%, 99%, and 99.9% confidence intervals, respectively.

**Figure 11:**
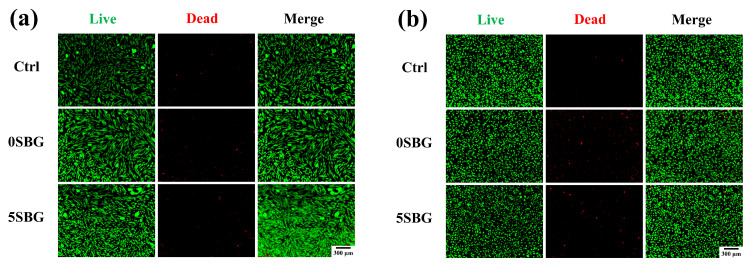
Live/dead staining assay of (**a**) L929 and (**b**) MG63 cell lines for 0SBG and 5SBG powders.

Two types of cell lines (L929 and MG63) were used to evaluate the biocompatibility of 650 °C-calcined SBG powders by using a cell counting kit-8 (CCK8) assay. The statistical results represent three independent experiments, and n = 3 for each experiment. Figure 10 shows the cell viability of ISO-required L929 and human osteosarcoma MG63 cell lines for all 0–7SBG powders. Most of the cell viabilities for both cells were larger than 100% (except 93.94% for 3SBG tested by L929), and all of them met the ISO10993-5 standard requirement of 70% [43]. Generally, the cell viability of MG63 was higher than that of L929. It is also interesting to note that 5SBG exhibited the highest cell viability for both the L929 (117.22 ± 6.65%) and MG63 (121.13 ± 2.13%) cell lines. This indicates that, though all the SBG exhibited good biocompatibility, the 5SBG powder was the best among them. According to the result of the statistical analysis, 5SBG has significant differences with the control group (*p*-value = 0.0019) and 3SBG (*p*-value = 0.0005) for the L929 cells. In addition, 5SBG was significantly different from the control (*p*-value = 0.0017) and 7SBG (*p*-value = 0.0221) for the MG63 cells. Selected samples (0SBG and 5SBG) were examined further by using the live/dead staining assay, and the results are shown in Figure 11. Good cell proliferation and growth with limited dead cells for both the L929 (Figure 11a) and MG63 (Figure 11b) cells were observed for the 0SBG and 5SBG powders. This confirmed the excellent biocompatibility of the so-prepared SBG powders [44]. The release of Sr^2+^ ion from bioactive glass powder has proven to play a main role in cell proliferation and differentiation and is recognized for promoting osteoblast activity [45]. Owing to Sr^2+^ ions, the 5SBG powder exhibited the largest cellular activity in both the L929 and MG63 cells. The cell viability results are summarized in Table 6.

**Figure 12:**
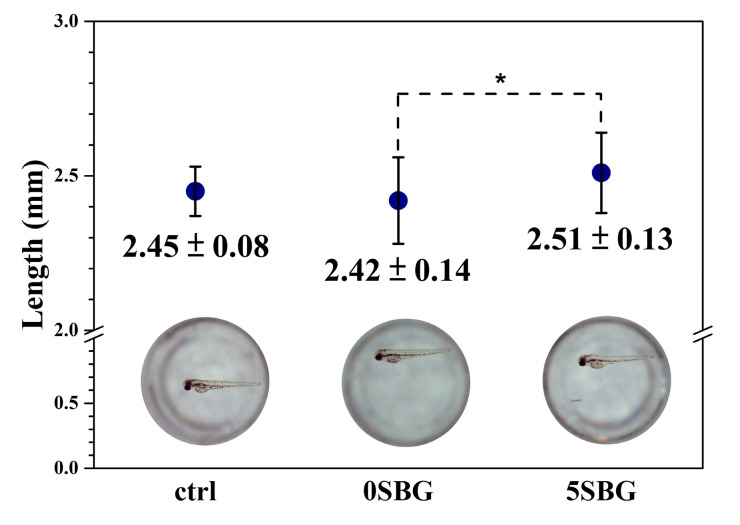
The average length and a typical image of zebrafish after 72 h post-fertilization. * indicated that these two samples were statistically different at a 95% confidence interval.

An in vitro animal test was performed using a zebrafish model (n = 30) to evaluate the cytotoxicity of the SBG powder. The hatch rate of the control group was 86.67%, and 0SBG and 5SBG had similar hatch rates of 83.33% and 86.57%. After hatching, the mortality rate was 0% for all three examined groups. Figure 12 shows the average length of the zebrafish after 72 h post-fertilization (i.e., hpf) and the images of selected ones from each group. The average length was 2.45 ± 0.08 mm for the control group, which remained the same for 0SBG (2.42 ± 0.14 mm), and slightly increased to 2.51 ± 0.13 mm for 5SBG. Only 0SBG and 5SBG were significantly different, with a *p*-value of 0.0294. This suggests that, in the zebrafish animal model, 5SBG did not exhibit cytotoxicity and was beneficial for the growth of zebrafish [46]. Bioactive glasses are known to release biologically active ions, such as calcium, silicon, and phosphorus, to promote skeletal development. The observed increase in body length may be attributed to the stimulatory effects of BG-derived ions on bone tissue formation and cellular proliferation [46]. Moreover, strontium is known to stimulate osteoblast activity to accelerate bone differentiation and mineralization during early development. The synergistic effect of Sr ions, alongside the release of Ca and Si from the BG matrix, [47] implies there is an osteoinductive potential in Sr-added bioactive glass when applied to zebrafish models.

The study by Moghanian et al. [21] reported bioactivity and biological studies by MC3T3-E1 of Sr-doped BG (0, 5, and 10 mol.%) prepared by the sol-gel process and calcined at 700 °C. The present study focused more on the effect of calcination temperature (400–650 °C) and the doping of Sr elements (0–7 mol.%). Biocompatibility was investigated by L929 and MG63 cells. In addition, an in vitro animal test using a zebrafish model was used for toxicological studies of SBG. From energy and cost considerations, sol-gelled bioactive glass powder calcined at 650 °C exhibited promising mineralization ability, biocompatibility, and non-cytotoxicity. Due to the limitations of the present work, further attempts to use SBG powder as bioactive materials to prepare bioscaffolds or compare the bioactive performance with commercial products are not available. The so-prepared SBG powder revealed its potential for bone regeneration applications. Based on the results, 5SBG may be the optimal additive for the preparation of bioscaffolds. Further investigations involving the preparation of SBG-added bioscaffolds, incorporation with BMP-2 growth factor, and comparison with commercially available bioscaffolds are in progress to determine whether this addition will improve bone formation in animal models. At present, the experiments in the current study demonstrate the potential clinical application of 5SBG-added composite bioscaffolds.

## 3. Conclusions

Sr-added 58S bioactive glass (0-7SBG) powders were prepared successfully using the sol-gel process, followed by calcination at various temperatures. Calcination at 650 °C for 3 h was able to prepare the desired bioactive glass powder. After calcination at 650 °C, all SBG powders possessed a major phase of Ca_14.92_(PO_4_)_2.35_(SiO_4_)_5.65_ that decreased with increasing amount of Sr addition. These SBG powders were mesoporous with an average pore size of ~6 nm. The 0SBG powder exhibited a specific surface area of 204.31 m^2^/g and increased to a maximum of 233.07 m^2^/g for 5SBG. The formation of hydroxyapatite was observed after immersing SBG powders in simulated body fluid. Deconvolution of FTIR spectra before and after immersion in SBF showed that the amount of HA formation was increased with Sr doping. The 5SBG powder exhibited good mineralization ability and a maximum of 76.7% for the amount of HA formation after 7 days of SBF immersion. All SBG powders were biocompatible, and the 5SBG powder was the best, with the highest cell viabilities of 117.22% and 121.13% for the L929 and MG63 cells, respectively. In the zebrafish animal model, the 0SBG and 5SBG powders were noncytotoxic, and the length of the zebrafish for the 5SBG group was the largest compared to that of the control or the 0SBG group. The 5SBG powder was optimal and exhibited potential to be used in bone tissue engineering.

## 4. Materials and Methods

### 4.1. Preparation of SBG Powder

Tetraethyl orthosilicate (TEOS, 98.0%), triethyl phosphate (TEP, 99%), and calcium nitrate tetrahydrate (Ca(NO_3_)_2_·4H_2_O, 99–103%) were used as the starting materials for the preparation of 58S bioactive glass. Partial substitution of calcium by strontium (0, 3, 5, and 7 mol%) was designed to modify the composition of bioactive glass, and strontium nitrate (Sr(NO_3_)_2_, ≥99.0%) was used as the source for strontium ions. The strontium-added bioactive glass powder was coded as 0-7SBG, respectively. Table 7 summarizes the chemical compositions of various Sr-added bioactive glass powders investigated in the present study. 1M HNO_3_ was added to distilled water at a ratio of 1:3 to prepare a solution of 200 mL and mixed by magnetic stirring at 300 rpm for 15 min at 37 °C. TEOS, TEP, Ca(NO_3_)_2_·4H_2_O, and Sr(NO_3_)_2_ were added sequentially to the HNO_3_-containing solution every 15 min. The final mixture solution was stirred for 4 h, kept in a 37 °C incubator for 3 days, and dried in a vacuum oven (80 °C) for 1 day. The as-prepared powders were then calcined at a temperature of 400, 500, 575, and 650 °C for 3 h, respectively.

### 4.2. Characterization of SBG Powder

To determine the suitable calcination temperature, the as-prepared dried SBG powder was examined using a thermogravimetric analyzer (TGA-50, Shimazu, Kyoto, Japan) to observe the weight change under an ambient atmosphere with a heating rate of 10 °C/min from room temperature to 800 °C. The thermogravimetric analyzer was calibrated using a certified reference standard, and the temperature error was within ±1 °C. The as-prepared and calcined SBG powders were examined using X-ray diffraction (XRD) to reveal the crystalline structure. An X-ray diffractometer (D2 PHASER, Bruker, Billerica, MA, USA) was operated at an accelerating voltage of 30 kV and a current of 10 mA. Cu Kα radiation (λ = 1.542 Å) was filtered by a Ni film and used to obtain the XRD 2θ pattern ranging from 15° to 75° at a step size of 0.05° with a scanning speed of 3.75°/min. The crystalline phases within XRD patterns were identified using an evaluation software (DIFFRAC.EVA, version 5.2., Bruker, Billerica, MA, USA). Only the top three major crystalline phases were used for calculation, and the error was less than 3%. The surface area, average pore diameter, pore size distribution, and total pore volume were measured according to the BET method using the surface area and porosity analyzer (TriStar II plus, Micromeritics, Norcross, GA, USA). The samples were degassed at 300 °C for 3 h. The analysis was performed in a N_2_ atmosphere, using adsorption–desorption isotherms at 77 K. The bonding of various functional groups for 0–7SBG powders was examined using Fourier transform infrared (FTIR) spectroscopy (Spectrum GX, Perkin-Elmer, Norwalk, CT, USA). The SBG powders were mixed with KBr, pressed into a disk, and examined using FT-IR to record the transmission spectra within a wavelength of 400–2000 cm^−1^ with a resolution of 4 cm⁻^1^, averaging eight scans per sample. The instrument was routinely calibrated according to the manufacturer’s instructions, and the error in peak position was within ±2 cm⁻^1^. The powder morphology of the SBG powder was further observed by a field emission scanning electron microscope (FE-SEM, SU-8200, Hitachi, Tokyo, Japan).

### 4.3. In Vitro Mineralization and Biocompatibility of SBG Powders

Selected SBG powders were immersed in simulated body fluids (SBFs) for different periods without replacing the solutions to simulate the mineralization reaction. SBF with similar ionic concentration as human plasma, composed of NaCl, NaHCO_3_, KCl, K_2_HPO_4_·3H_2_O, MgCl_2_·6H_2_O, CaCl_2_, and Na_2_SO_4_, was prepared according to the literature [48]. All of these powders were dissolved in distilled water, and pH values were adjusted to 7.4 at 37 °C using TRIS and HCl. The immersed SBG powders were examined using XRD, FT-IR, and SEM to determine the bioactivity of SBG powders.

For the biocompatibility test, SBG powder extracts with a weight-volume ratio of 20 mg/mL were immersed in Dulbecco’s modified Eagle’s medium (DMEM, (Cytiva, Hyclone, South Logan, UT, USA) with 10% fetal bovine serum (FBS, Cytiva, Hyclone, South Logan, UT, USA) and 1% antibiotic/antimycotic solution (Cytiva, Hyclone, South Logan, UT, USA) for 24 h. Fibroblast cell L929 (ISO standard) and osteoblast cell MG 63 were seeded at 1 × 10^4^ cells/well in 96-well plates and cultured for 24 h in a humidified atmosphere of 5% CO_2_ at 37 °C. After the cells were attached, DMEM was replaced with the SBG extracts. After 1 day of incubation, 10 µL of cell counting kit-8 solution (CCK-8, Sigma-Aldrich, St. Louis, MO, USA) was added into each well and incubated for another 2 h. The biocompatibility was measured at 450 nm using a Multiskan™ FC Microplate Photometer (Thermo-Fisher, Waltham, MA, USA). The relative OD450 (optical density at 450 nm) ratio was normalized to the control group, and the cell viability was presented as the mean ± SD in percentage. The live/dead cell staining of L929 and MG63 (using LIVE/DEAD™ Cell Imaging Kit (488/570), Invitrogen, Carlsbad, CA, USA) incubated with SBG extract were examined (Green—live cells, Red—dead cells) and the morphology of cells was observed using a fluorescence microscope (PAULA Smart Cell Imager, Leica Microsystems GmbH, Wetzlar, Germany).

The cytotoxicity of SBG powders was evaluated using a zebrafish animal model [46]. Adult zebrafish (Danio rerio, AB strain) were reared at 28 °C with 14 light—10 dark photoperiods. The temperature (28 ± 0.5 °C) and pH (7.0 ± 0.5) of the water were monitored and controlled. AB wild-type (WT) strains of zebrafish were provided by the Taiwan Zebrafish Core Facility of Academia Sinica (Taipei, Taiwan). The SBG powders were immersed in artificial normal water for 1 day, and 1000X extraction was prepared as an experimental medium. Three test groups (control, 0SBG, and 5SBG) were examined at the same time, and each group contained 30 embryos. The hatch rate, mortality rate, and the fish length, after 72 h post-fertilization, of each group were recorded for comparison. The experimental protocols were approved by the Taipei Medical University Animal Care and Utilization Committee (approval no.: LAC-2024-0022).

## Figures and Tables

**Table 1:** Results of XRD analysis of 0SBG powder after calcination at various temperatures.

Sample	Composition	Percentage (wt.%) *	PDF Card No
As prepared	amorphous	100%	-
400 °C	Ca_9_HPO_4_(PO_4_)_5_OH Ca_2_SiO_4_ CaCO_3_	47.5% 39.4% 13.0%	PDF 46-0905 PDF 29-0369 PDF 05-0586
500 °C	Ca_2_SiO_4_ Ca_9_HPO_4_(PO_4_)_5_OH CaCO_3_	47.4% 45.9% 6.6%	PDF 29-0369 PDF 46-0905 PDF 05-0586
575 °C	Ca_10_(PO_4_)_6_O	100%	PDF 89-6495
650 °C	Ca_14.92_(PO_4_)_2.35_(SiO_4_)_5.65_	100%	PDF 83-1494

* Semi-quantitative analysis of crystalline phase(s) by DIFFRAC-EVA software. The amount of amorphous phase was not included.

**Table 2:** Results of XRD analysis of 5SBG powder after calcination at various temperatures.

Sample	Composition	Percentage (wt.%) *	PDF Card No
As prepared	Ca_0.33_Sr_0.67_(NO_3_)_2_ Sr(NO_3_)_2_	69.4% 35.1%	PDF 26-1073 PDF 25-0746
400 °C	Ca_0.33_Sr_0.67_(NO_3_)_2_ Ca_2_SiO_4_	86.0% 14.0%	PDF 26-1073 PDF 29-0369
500 °C	Ca_0.33_Sr_0.67_(NO_3_)_2_ Ca_2_SiO_4_ Ca_2_P_2_O_7_	59.0% 25.2% 15.8%	PDF 26-1073 PDF 29-0369 PDF 33-0297
575 °C	Ca_14.92_(PO_4_)_2.35_(SiO_4_)_5.65_ Ca_2_SiO_4_ Ca_2_P_2_O_7_	45.6% 43.0% 11.4%	PDF 83-1494 PDF 29-0369 PDF 33-0297
650 °C	Ca_14.92_(PO_4_)_2.35_(SiO_4_)_5.65_ Sr_2_SiO_4_	87.8% 12.2%	PDF 83-1494 PDF 38-0271

* Semi-quantitative analysis of crystalline phase(s) by DIFFRAC-EVA software.

**Table 3:** Results of XRD analysis of 0–7SBG powder after calcination at 650 °C for 3 h.

Sample	Composition	Percentage (wt.%) *	PDF Card No
0SBG	Ca_14.92_(PO_4_)_2.35_(SiO_4_)_5.65_	100%	PDF 83-1494
3SBG	Ca_14.92_(PO_4_)_2.35_(SiO_4_)_5.65_ Sr_2_SiO_4_	91.5% 8.5%	PDF 83-1494 PDF 38-0271
5SBG	Ca_14.92_(PO_4_)_2.35_(SiO_4_)_5.65_ Sr_2_SiO_4_	87.8% 12.2%	PDF 83-1494 PDF 38-0271
7SBG	Ca_14.92_(PO_4_)_2.35_(SiO_4_)_5.65_ Ca_2_SiO_4_ Sr_2_SiO_4_	49.9% 42.9% 7.2%	PDF 83-1494 PDF 29-0369 PDF 38-0271

* Semi-quantitative analysis of crystalline phase(s) by DIFFRAC-EVA software.

**Table 4:** BET results of various SBG powders after calcination at 650 °C for 3 h.

	Sample Code	0SBG	3SBG	5SBG	7SBG
Property					
BET Surface Area (m^2^/g)		204.31 ± 0.55	216.41 ± 1.15	233.07 ± 1.13	232.56 ± 1.27
Average Pore Diameter (nm)		6.04	6.39	5.86	5.92
Total Pore Volume (cm^3^/g)		0.32	0.32	0.31	0.31

**Table 5:** The fitting area amount of PO_4_^3−^ and CO_3_^2−^ groups for 0~7SBG before and after 7 days immersion in simulated body fluid.

	Before		After	
Sample	PO_4_^3−^	CO_3_^2−^	PO_4_^3−^	CO_3_^2−^
0SBG	56.8%	8.6%	77.2%	1.5%
3SBG	50.2%	6.5%	70.3%	0.7%
5SBG	43.7%	5.9%	77.2%	1.1%
7SBG	44.8%	2.4%	66.0%	1.4%

**Table 6:** Cell viability results of L929 and MG63 for SBG powders.

	Sample	Control	0SBG	3SBG	5SBG	7SBG
Cell						
L929		100.00 ± 5.99	106.04 ± 0.90	93.94 ± 2.26	117.22 ± 6.65	105.04 ± 3.95
MG63		100.00 ± 7.97	110.93 ± 7.26	111.89 ± 4.46	121.13 ± 2.13	102.10 ± 5.46

**Table 7:** Chemical compositions of various Sr-added bioactive glass powders.

Sample Code	Bioactive Glass Composition (mol%)			
	SiO_2_	CaO	P_2_O_5_	SrO
0SBG	60	36	4	0
3SBG	60	33	4	3
5SBG	60	31	4	5
7SBG	60	29	4	7

## Data Availability

The original contributions presented in this study are included in the article/Supplementary Material. Further inquiries can be directed to the corresponding authors.

## Supplementary Materials

The following supporting information can be downloaded at: https://www.mdpi.com/article/10.3390/gels11060401/s1, Figure S1: X-ray diffraction patterns of as-prepared and calcined (a) 0SBG and (b) 5SBG powder; Figure S2: X-ray diffraction patterns of 0-7 SBG powder after calcination at 650 °C for 3h.; Figure S3: (a) 0SBG, (b) 3SBG, (c) 5SBG, and (d) 7SBG powder XRD patterns with their observed crystallin phases and structures after immersion in simulated body fluid for 1, 3, and 7 days; Figure S4: The mapping of Si, Ca, P, Sr, C, and O elements on (a) 0SBG, (b) 0SBG-7D, (c) 5SBG, and (d) 5SBG-7D powders; Figure S5: pH value of 0SBG (black square symbol) and 5SBG (red circle symbol) in different SBF immersion times (0, 1, 3, and 7 days); Figure S6: Sr^2+^ concentration of 5SBG after immersion in SBF for different period of time; Table S1: Comparison of various sol-gelled 58S bioactive glass powder with Sr addition; Table S2: EDX of 0SBG and 5SBG before and after 7 days.
