# Supplementary material for: Effect of Calcination Temperature and Strontium Addition on the Properties of Sol-Gelled Bioactive Glass Powder
Source: Gels. 2025 May 27;11(6):401. doi: 10.3390/gels11060401 (PMC12191557; doi:10.3390/gels11060401)
Supplement: Supplementary file 1 [file gels-11-00401-s001.zip › gels-3655686-supplementary.pdf]

Table S1 summarizes the 58S bioactive glass powder prepared by sol-gel process. The substitution of Ca by Sr was usually less than 10 mol%. Calcination was performed at 700 or 800°C with various durations.

Table S1: Comparison of various sol-gelled 58S bioactive glass powder with Sr addition.

**Bioactive glass composition**	**Sr addition**	**Calcination Parameters**	**Important findings**	**Ref.***
(38-x) CaO–xSrO–58SiO_2–4P_2O_5	2,4,6,8,10 mol%	700°C for 2h	● Decreased surface area and pore volume<br>● Delay the initial formation of hydroxyapatite, and the formed HA had reduced crystallinity with increasing SrO	[4]
60 SiO_2–(36-x) CaO–4 P_2O_5–x SrO	0,5,10 mol%	700°C for 3h	● 5% SrO increased both differentiation and proliferation of MC3T3-E1 cells<br>● 10% SrO resulted in a decrease in bioactivity	[21]
60 SiO_2–(34-x) CaO–4 P_2O_5–x SrO	2,4,6,8 mol%	800 °C for 3 h	● the sample S6 was selected as the optimum sample in terms of bioactivity and cell proliferation that can be potentially used for drug delivery systems as well as orthopedic and dental applications	[22]
60SiO_2–(36-x) CaO-4 P_2O_5-x	9,18,27,36 mol%	700 °C for 5 h	● With increasing amounts of SrO substituted in the place of CaO, the materials showed phase separation with the formation of amorphous and crystalline phases, strontium apatite ($Sr_5(PO_4)_3OH$) and strontium silicate (Sr_2SiO_4)<br>● At higher substitutions (75 and 100%SrO for CaO), HA nucleation was not found to occur this may have been due to low amount of phosphate released from the original glass–ceramic as a result of it being locked up in the strontium apatite phase	[23]

*Referred to the reference in the main text.

The detailed information of crystalline phases and structures on different calcined temperature XRD patterns were summarized in Figure S1.

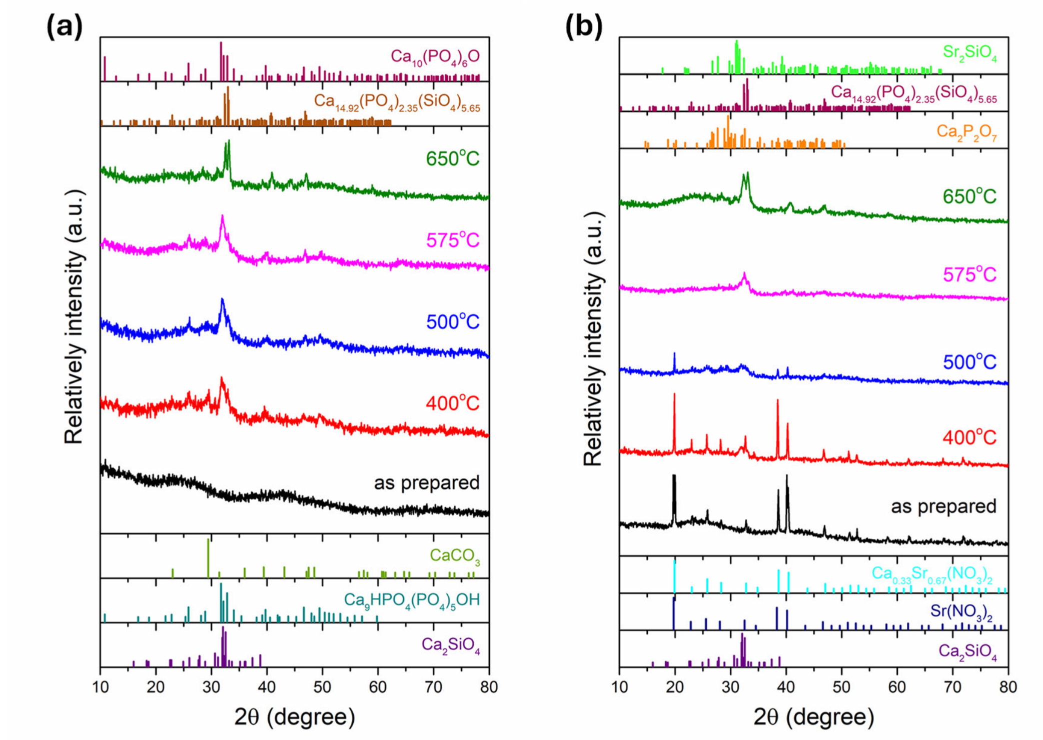

Figure S1: X-ray diffraction patterns of as-prepared and calcined (a) 0SBG and (b) 5SBG powder.

The different Sr doping amount of SBG XRD patterns with their detailed corresponding crystalline phases and structures were shown in Figure S2.

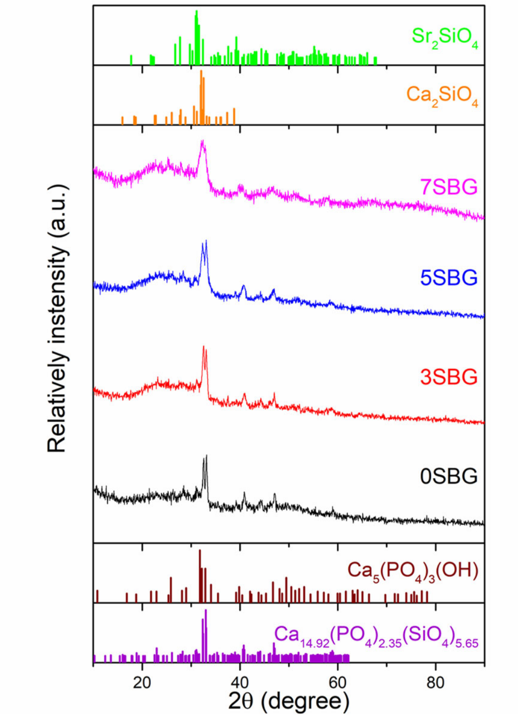

Figure S2: X-ray diffraction patterns of 0-7 SBG powder after calcination at 650°C for 3h.

Figure S3 showed the XRD patterns of 0~7SBG before and after immersion in SBF for different days (1, 3, and 7 days). The obtained structures were performed at the top and down in the patterns.

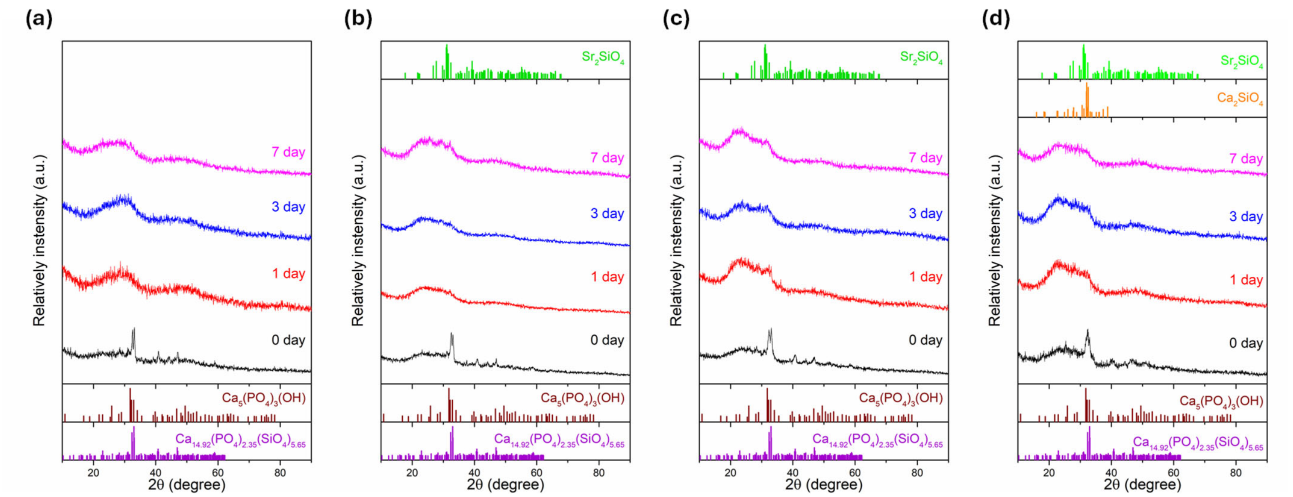

Figure S3: (a) 0SBG, (b) 3SBG, (c) 5SBG, and (d) 7SBG powder XRD patterns with their observed crystallin phases and structures after immersion in simulated body fluid for 1, 3, and 7 days.

Figure S4 shows elemental mappings of Si, Ca, P, Sr, C, and O elements for 0SBG and 5SBG before and after immersion in SBF for 7 days.

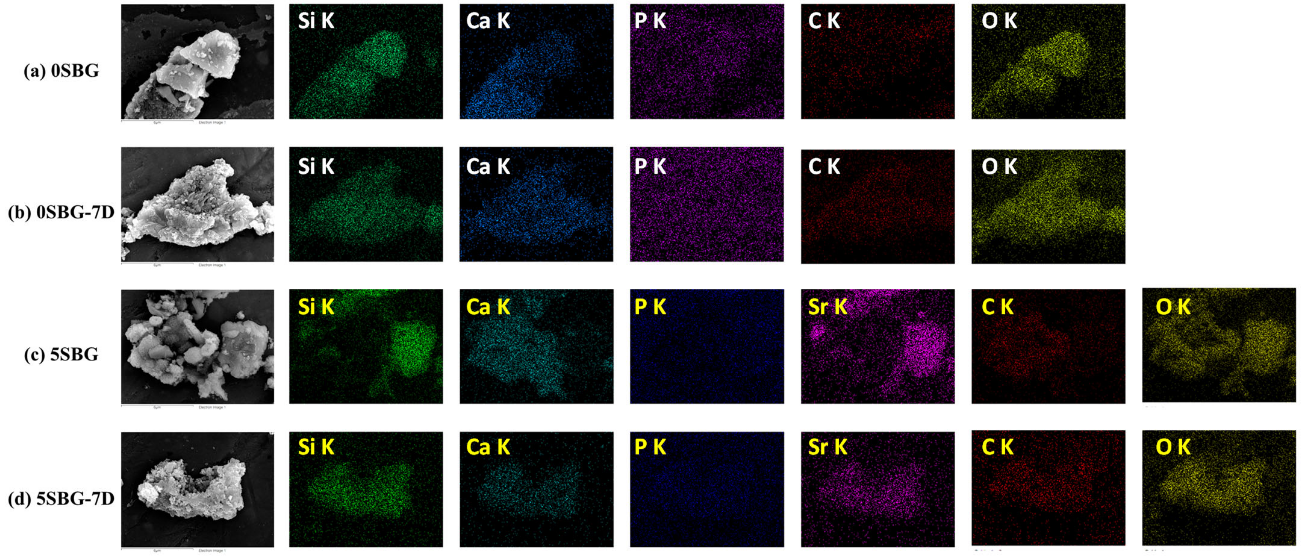

Figure S4: The mapping of Si, Ca, P, Sr, C, and O elements on (a) 0SBG, (b) 0SBG-7D, (c) 5SBG, and (d) 5SBG-7D powders.

The semi-quantitative analysis of 0SBG(-7D) and 5SBG(-7D) were obtained by EDX (Fig. S4), and the results were summarized in Table S2.

Table S2: EDX of 0SBG and 5SBG before and after 7 days.

	Element	Si K	Ca K	P K	Sr L	C K	O K	Totals
**0SBG**	Weight%	16.87	19.43	3.89	-	10.17	49.65	100
	Atomic%	11.64	9.39	2.44	-	16.4	60.13	
**0SBG-7D**	Weight%	12.36	11.4	3.32	-	20.04	52.89	100
	Atomic%	7.58	4.9	1.84	-	28.74	56.94	
**5S**	Weight%	12.78	18.6	1.6	6.65	12.63	47.73	100
	Atomic%	8.96	9.13	1.02	1.49	20.7	58.7	
**5SBG-7D**	Weight%	12.06	9.25	4.19	9.07	19.09	46.34	100
	Atomic%	7.98	4.29	2.51	1.92	29.51	53.79	

A pH meter was used to measure the pH value of 0SBG and 5SBG at room temperature in different SBF immersion times, Fig. S5.

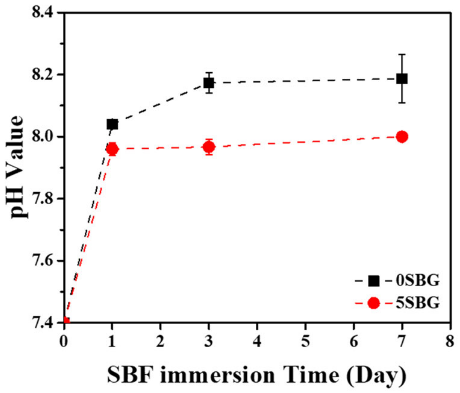

Figure S5: pH value of 0SBG (black square symbol) and 5SBG (red circle symbol) in different SBF immersion times (0, 1, 3, and 7 days).

ICP-OES was employed to measure the Sr^{2+} ion released after immersion in SBF for 1, 3, and 7 days, Fig. S6.

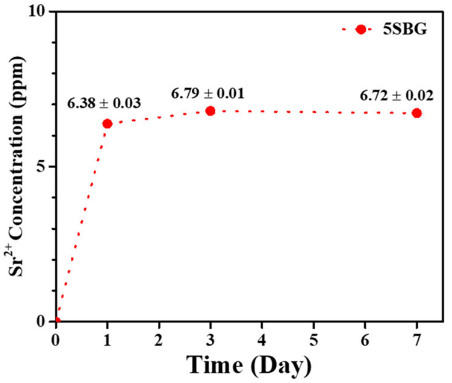

Figure S6: Sr^{2+} concentration of 5SBG after immersion in SBF for different period of time.
